# *Mucilaginibacter florum* sp. nov., isolated from the flower of *Coreopsis grandiflora* and *Mucilaginibacter oryzagri* sp. nov., isolated from rice paddy soil in Korea

**DOI:** 10.71150/jm.2509014

**Published:** 2025-12-31

**Authors:** Parthiban Subramanian, Jun Heo, Daseul Lee, Seunghwan Kim, Hyorim Choi, Yunhee Choi, Yiseul Kim

**Affiliations:** 1 Plant Disease Control Division, National Institute of Agricultural Sciences, Rural Development Administration, Wanju 55365, Republic of Korea; 2 Agricultural Microbiology Division, National Institute of Agricultural Sciences, Rural Development Administration, Wanju 55365, Republic of Korea; 3 Departmentof Biotechnology, Jeonbuk National University, Iksan 54596, Republic of Korea; 4 Departmentof Biological Sciences, Jeonbuk National University, Jeonju 54896, Republic of Korea

**Keywords:** polyphasic taxonomy, novel bacteria, flower, *Coreopsis grandiflora*, soil, plant-associated

## Abstract

Two aerobic, Gram-stain-negative, non-motile and rod-shaped bacterial strains designated GGG-R5^T^ and M4-18^T^ were isolated from flowers of golden wave (*Coreopsis grandiflora*) and rice paddy soil, respectively in the Republic of Korea. Both strains were pigmented and produced flexirubin-type pigments. Based on phylogenetic analysis using 16S rRNA gene sequence, both strains were placed within the genus *Mucilaginibacter* with *M. agri* R11^T^ and *M. jinjuensis* YC7004^T^ both being the closest relatives to GGG-R5^T^ (97.7%) and in case of M4-18^T^, *M. ginsenosidivorax* KHI28^T^ (98.5%) was the nearest neighbor. Characteristic to genus *Mucilaginibacter*, the major cellular fatty acids in both strains were iso-C_15:0_, iso-C_17:0 3-OH_, summed feature 3 (C_16:1_ *ω7c* and/or C_16:1_ *ω6c*); menaquinone-7 was the major menaquinone and phosphatidylethanolamine was the major polar lipid observed. Comparison of genome sequences with the other members of *Mucilaginibacter* indicated orthologous average nucleotide identity (orthoANI) at 73.3–73.5% for GGG-R5^T^ and 78.9–88.5% for M4-18^T^. Digital DNA-DNA hybridization (dDDH) values ranged at 19.1–19.7% between GGG-R5^T^ and its neighbor species. In case of M4-18^T^, the observed range was at 21.9–36.6%. Considering the 16S rRNA similarity, orthoANI and dDDH values as well as comparison of phenotypic and chemotaxonomic characteristics indicated that both strains belonged to genus *Mucilaginibacter* but were distinctly distinguishable from previously described species. The strains GGG-R5^T^ and M4-18^T^, therefore represent distinct novel species for which names *Mucilaginibacter florum* GGG-R5^T^ and *Mucilaginibacter oryzagri* M4-18^T^ are proposed. The type strains are GGG-R5^T^ (= KACC 22063^T^ = JCM 36590^T^) and M4-18^T^ (= KACC 22773^T^ = JCM 35894^T^).

## Introduction

The genus *Mucilaginibacter* was first described in 2007 with isolates originating from acidic *Sphagnum* peat bog (Pankratov et al., 2007). The genus was assigned to the family *Sphingobacteriaceae*, class *Sphingobacteriia* in the phylum *Bacteroidota*. Currently, there are 81 validly published species of *Mucilaginibacter* according to the List of Prokaryotic names with Standing in Nomenclature (LPSN) (Parte et al., 2020). The description of characteristics of the genus Mucilaginbacter was emended several times since its initial description in 2007 (Baik et al., 2010; Chen et al., 2014; Kim et al., 2022; Urai et al., 2008). Based on the recent description, species of *Mucilaginibacter* are Gram-stain-negative, aerobic or facultatively anaerobic, non-spore-forming, non-motile and (short) rod-shaped, exhibiting production of flexirubin-like pigment and exopolysaccharides. The predominant respiratory quinone is menaquinone-7 (MK-7) and the major fatty acids include iso-C_15:0_, iso-C_17:0_ 3-OH and summed feature 3 (C_16:1_ *ω7c* and/or C_16:1_ *ω6c*). Phosphatidylethanolamine (PE) is the major polar lipid and the genomes indicate a G + C content at a range of 39.1–49.8% (Kim et al., 2020, 2022; Pankratov et al., 2007).

Ecologically, a significant number of *Mucilaginibacter* species have been described to be originating from soil and freshwater environments (54 of the 81 validly published species in the genus) (Baik et al., 2010; Joung et al., 2014, 2015; Kang et al., 2021; Kim et al., 2013, 2020, 2022; Seo et al., 2020; Urai et al., 2008; Zheng et al., 2016). There are also species that tolerate several extreme conditions reported from cold as well as marine environments (Kang et al., 2013; Kim et al., 2022; Yoon et al., 2012; Zheng et al., 2016). A significant number of phyto-associated species have also been described from environments such as tree barks, stem, leaves and rotten wood (Aydogan et al., 2016, 2017; Kämpfer et al., 2014; Kang et al., 2021; Khan et al., 2013a, 2013b; Zhang et al., 2019). Previous reports have indicated that genomes of *Mucilaginibacter* species harbor genes that help them to well-adapt to their environments such as metal binding chaperones in case of *Mucilaginibacter kameinonensis* and *M. rubeus* isolated from gold and copper mines and DNA-repair as well as cold-stress response genes in several *Mucilaginibacter* species from the Arctic tundra soil (Kumar et al., 2025; Li et al., 2018). In the course of exploring the beneficial bacteria in agricultural environments, we isolated two strains of *Mucilaginibacter*, GGG-R5^T^ from flower and M4-18^T^ from soil in the Republic of Korea. The present study aimed to elucidate the exact taxonomic position of strains GGG-R5^T^ and M4-18^T^ by using a polyphasic approach based on the combination of phenotypic and genotypic characteristics.

## Materials and Methods

### Habitat and isolation

Flowers of golden wave (*Coreopsis grandiflora*) collected in Jeonju (35° 49' 46" N 127° 02' 18" E) and soil samples collected from rice paddy soil after rice cultivation in Miryang, Republic of Korea (35° 30' 44" N 128° 42' 38" E). The soil sample was composed of four subsamples taken from 20 × 20 cm^2^ at a depth of 20–30 cm. Entire flowers were suspended in sterile 0.85% (w/v) NaCl solution and shaken at 200 rpm on a rotary shaker for 30 min. Same protocol was also used for soil samples where samples were thoroughly mixed by shaking at 200 rpm on a rotary shaker for 30 min. For isolation of bacteria, the suspensions were serially diluted, spread on Reasoner’s 2A (R2A) agar (BD Difco) and aerobically incubated at 28°C for 72 h. At the end of incubation period, the strains GGG-R5^T^ and M4-18^T^ were isolated from the flower and soil suspensions, respectively. Preliminary, 16S rRNA gene sequence analysis was carried out for both strains. To further investigate their characteristics and compare with their closest phylogenetic relatives, *Mucilaginibacter agri* KACC 21228^T^, *Mucilaginibacter jinjuensis* KACC 16571^T^, *Mucilaginibacter polytrichastri* KACC 19030^T^, *Mucilaginibacter straminoryzae* KACC 24163^T^, *Mucilaginibacter ginsenosidivorax* KACC 14955, *Mucilaginibacter pineti* KACC 23032^T^, *Mucilaginibacter flavus* KACC 18090^T^, and *Mucilaginibacter dorajii* KACC 14556^T^ were acquired from the Korean Agricultural Culture Collection (KACC), Republic of Korea. Strains GGG-R5^T^ and M4-18^T^ were then subjected to phenotypic, biochemical, physiological, enzymatic analyses, taxonomic marker and genome based comparison for combined polyphasic taxonomic studies.

### Phenotypic characterization

Cell morphology of strains GGG-R5^T^ and M4-18^T^ were examined by transmission electron microscopy (LEO 912AB, LEO Electron) and phase contrast light microscopy (Axio Imager A1, Carl Zeiss) using cells grown on R2A at 28°C for 2 days. Gram staining was carried out using commercial Gram staining kit (Sigma Korea) according to the manufacturer’s instructions. Oxidase activity was checked using 1% (w/v) tetramethyl-*p*-phenylenediamine, and catalase activity using 3% (w/v) H_2_O_2_, observing for effervescence. Growth at different temperatures (4, 10, 15, 20, 25, 28, 30, 35, 40, and 45°C) and various pH conditions (pH 4.0–10.0, at intervals of 1.0 pH unit) was assessed in R2A broth after 7 days of incubation at 28°C. For growth under different pH conditions, the pH of the medium was adjusted with 20 mM citrate-phosphate buffer (pH 4.0–7.0), 20 mM Tris-hydrochloride buffer (pH 8.0–9.0) and 20 mM carbonate-bicarbonate buffer (pH 10.0). Salt tolerance was assessed in R2A broth supplemented with NaCl (0–9% in increments of 1%, w/v). Hydrolysis of casein (1%, w/v), CM-cellulose (0.5%, w/v), chitin (1.0%, w/v), hypoxanthine (0.5%, w/v), starch (1%, w/v), Tween 80 (1%, w/v) and xanthine (0.5%, w/v) was examined using R2A supplemented with each substrate. Growth under anaerobic conditions was assessed by incubating in a Gas Pak anaerobic system at 28°C for 7 days. Motility was observed on R2A solidified with 0.3% agar. The physiological and biochemical characteristics were tested using API ZYM, API 20NE, API 32GN, and API 50CH test kits (bioMérieux, France). For API ZYM incubation was carried out for 5 h at 28°C. In case of API 20NE, API 32GN, and API 50CH results were recorded after 7 days of incubation at 28°C. Production of flexirubin-type pigment was confirmed using earlier reported protocols (Reichenbach, 1989).

### 16S rRNA gene based phylogeny

Amplification of the 16S rRNA gene was performed using universal primers 27F and 1 492R, followed by sequencing at Solgent, Daejon, Republic of Korea (Lane, 1991). For the 16S rRNA gene based phylogenetic analyses, initially the obtained 16S rRNA gene sequences were identified using EzBioCloud servers (Yoon et al., 2017a) and sequences of their closely related species obtained. Phylogenetic trees were constructed using MEGA 11 (Tamura et al., 2021) based on the neighbor-joining (NJ) (Saitou and Nei, 1987), maximum-likelihood (ML) (Felsenstein, 1981) and maximum-parsimony (MP) (Fitch, 1971) algorithms, and tree topologies evaluated by the bootstrap resampling method of Felsenstein (Felsenstein, 1985) with 1,000 replicates. Evolutionary distances for the NJ and ML analyses were estimated by the algorithm from the Kimura’s two-parameter model (Kimura, 1980).

### Chemotaxonomic characterization

To study the cellular fatty acid methyl ester composition, strains GGG-R5^T^ and M4-18^T^ and the reference strains were cultivated on nutrient agar (NA) medium at 28°C for 24–48 h (depending on strains), followed by fatty acid methyl ester (FAME) analysis using Sherlock Microbial Identification System (MIDI) (Richter and Rosselló-Móra, 2009; Sasser, 1990). For the analysis of polar lipids and isoprenoid quinone, cells were obtained after cultivation on R2A at 28°C for 24 h. Extraction of polar lipids and isoprenoid quinone was performed as described by Minnikin et al. (1984). Polar lipids were separated using two-dimensional thin-layer chromatography on silica gel 60 G plates (Merck) and detected by spraying with molybdophosphoric acid for total lipids, ninhydrin for free amino groups, molybdenum blue for phosphorus-containing lipids, *α*-naphthol sulfuric acid for glycolipids and Dragendorff’s solution for quaternary nitrogen. The crude quinone solution was purified using Sep-Pak Silica Vac cartridges (Waters) and analyzed by reverse-phase high-performance liquid chromatography (Waters) as described previously (Hiraishi et al., 1996).

### Genomic characterization

Genomic DNA was extracted from both strains using a Maxwell^®^ RSC Tissue DNA Extraction Kit (Promega, Korea). Pacbio Sequel-Ⅱ sequencing platform (Pacific Biosciences, USA) and Illumina NovaSeq6000 150PE instrument (Illumina, USA) at Macrogen (Republic of Korea) were used for whole genome sequencing of both strains. The sequence reads were assembled using HiFiasm v. 0.16.0 where PacBio long-read data was preliminarily used and Illumina reads were used to resolve gaps or repetitive regions. Genome completeness analysis using BUSCO (Manni et al., 2021) and annotation based on Prokka (Seemann, 2014) were carried out using the Galaxy online server (The Galaxy Community, 2022). Further, genomes of strains GGG-R5^T^ and M4-18^T^ were annotated using the Rapid Annotation Subsystem Technology (RAST) version 2.0 (Brettin et al., 2015) and the NCBI prokaryotic genome annotation pipeline (PGAP) (Tatusova et al., 2016). The orthologous average nucleotide identity (OrthoANI) values (%) were calculated using OrthoANI in EzBioCloud (Yoon et al., 2017b). For other genome based taxonomic analyses, the genome sequence data was uploaded to the Type (Strain) Genome Server (TYGS) (Meier-Kolthoff and Göker, 2019). Information on nomenclature, synonymy and associated taxonomic literature was provided by TYGS's sister database, LPSN (Meier-Kolthoff et al., 2022). Genome-to-genome distance (GGDC) analysis was carried out using the GGDC calculator (Meier-Kolthoff et al., 2022). digital DNA:DNA hybridization (dDDH) was carried out as part of TYGS analyses and the values of d4 formula which calculates sum of all identities found in high-scoring segment pairs (HSPs) divided by overall HSP length reported independent of the genome length was reported. Genome based tree from TYGS was drawn with FastME 2.1.6.1 using Genome Blast Distance Phylogeny (GBDP) distances calculated from genome sequences and branch lengths were scaled in terms of GBDP distance formula d5. For genome derived bacterial core genes-based phylogenetic analysis, 92 up-to-date bacterial core genes were extracted from the genomes of neighboring species based on TYGS analyses followed by multiple-alignment, and construction of a phylogenetic tree using the up-to-date bacterial core gene (UBCG) tool ver. 3 (Na et al., 2018).

For functional genome analysis, protein sequences were analyzed using eggNOG-mapper v2 against the eggNOG v5.0 database (Cantalapiedra et al., 2021; Huerta-Cepas et al., 2019). Genes coding for secondary metabolite were identified using antiSMASH v7.0 (Blin et al., 2023). Resistome sequences in the genomes were searched using the Resistance Gene Identifier (RGI) web portal (Alcock et al., 2023). As strains M4-18^T^ and GGG-R5^T^ were isolated from agricultural environments, the genomes were further studied using plant interaction factors prediction tool (PIFAR-Pred) and plant growth-promoting traits prediction tool (PGPT-Pred) hosted at the PLant-associated BActeria web resource (PLaBAse), University of Tübingen (Patz et al., 2021).

### Nucleotide sequence accession numbers

The GenBank accession numbers for the 16S rRNA gene sequences of strains GGG-R5^T^ and M4-18^T^ are PP236896 and OP377360, respectively. The NCBI accession numbers for the whole-genome sequences of strains GGG-R5^T^ and M4-18^T^ are CP117877 and CP117883, respectively.

## Results and Discussion

### 16S rRNA gene based phylogeny

**Fig. 1.**
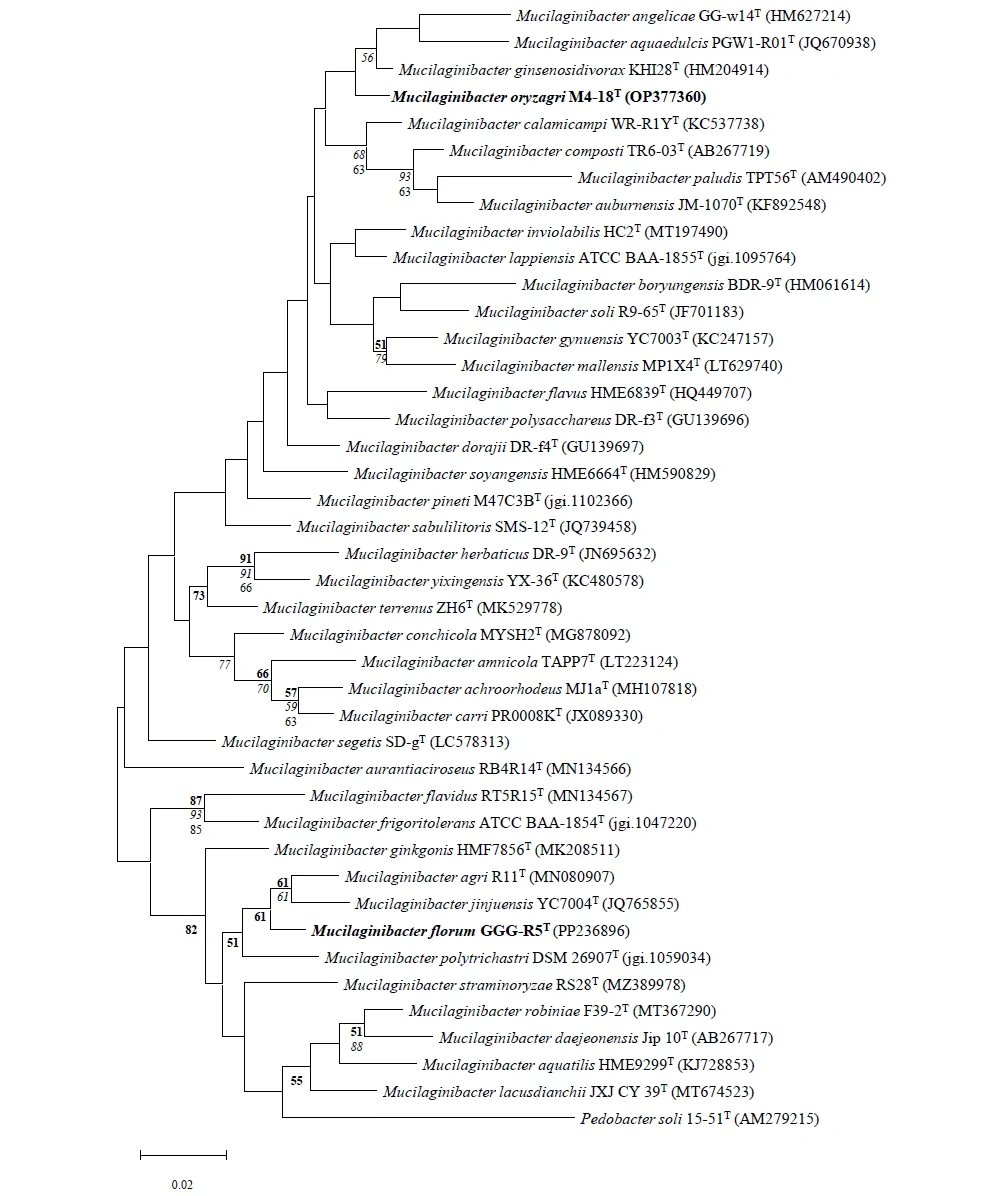
Maximum-likelihood trees of strains GGG-R5^T^ and M4-18^T^ (in bold) along their nearest neighbors based on 16S rRNA gene sequence comparisons. NCBI GenBank accession numbers of the 16S rRNA gene sequences are given in parentheses. Bootstrap values (≥ 50%) based on 1,000 replicates are shown at the branch nodes. *Pedobacter soli* 15-51^T^ was used as an outgroup. Bootstrap values from the NJ and MP analyses on matching corresponding nodes are given in italics and regular text respectively next to the bootstrap support of ML analyses (given in bold).

Preliminary 16S rRNA gene sequence analysis indicated that strains M4-18^T^ and GGG-R5^T^ were putatively novel (< 98.5% similarity) and belonged to the genus *Mucilaginibacter*. The assembled 16S rRNA gene sequences were submitted to the NCBI GenBank with accession numbers PP236896 and OP377360, for strains GGG-R5^T^ and M4-18^T^, respectively. Strain GGG-R5^T^ was found to be most closely related with *M. agri* R11^T^ and *M. jinjuensis* YC7004^T^ (97.7%) and in case of strain M4-18^T^, *M. ginsenosidivorax* KHI28^T^ (98.5%) was found to be the nearest neighbor. The phylogenetic trees indicated that strain GGG-R5^T^ was placed in a distinct clade within the genus *Mucilaginibacter* along with *M. agri* R11^T^, *M. jinjuensis* YC7004^T^, and *M. polytrichastri* DSM26907^T^, whereas strain M4-18^T^ was placed along with *M. ginsenosidivorax* KHI28^T^, *M. dorajii* JCM 16601^T^, and *M. flavus* KACC 18090^T^ (Fig. 1).

### Phenotypic characterization

Strains GGG-R5^T^ and M4-18^T^ grew aerobically with rod-shaped and were Gram-stain-negative. Motility tests indicated both strains were non-motile. Both catalase as well as oxidase activities were positive (Table 1). Strain GGG-R5^T^ measured at 0.6–0.65 × 1.2–2.5 μm and strain M4-18^T^ at 0.6–0.7 × 2.0–8.0 μm (Fig. S1). On R2A agar, colonies of strain GGG-R5^T^ were light pink, convex, round and watery, and measured at 2–3 mm after 2 days of incubation at 28°C. Colonies of strain M4-18^T^ were milky with a yellow tinge and irregular with a diameter smaller than 0.5 mm after 2 days of incubation at 28°C. For strain GGG-R5^T^, growth was observed at 4–37°C (no growth at 40°C, optimum 28°C), pH 5.0–9.0 (optimum pH 7.0–8.0) and 0–1% (w/v) NaCl (no growth at 2%, optimum 0%). In case of strain M4-18^T^, growth was observed at 4–35°C (no growth at 37°C, optimum 28°C), pH 5.0–8.0 (optimum pH 7.0) and 0–1% (w/v) NaCl (no growth at 2%, optimum 0%).

Strain GGG-R5^T^ was positive for hydrolysis of casein, but negative in case of strain M4-18^T^. Both strains could not hydrolyze CM-cellulose, chitin, hypoxanthine, starch, Tween 80 and xanthine. The differential characteristics of the two strains from other *Mucilaginibacter* species were evident during the analysis of their biochemical characteristics. Results from the assays followed by comparison with their nearest neighbors indicated that strains GGG-R5^T^ and M4-18^T^ were consistent in characteristics such as nitrate reduction, indole production, fermentation of glucose, aesculin hydrolysis and arginine dihydrolase as well as *β*-galactosidase activity (Tables 1 and S1). However, differential results were observed for both strains in case of gelatin hydrolysis. Comparison of enzyme activities using API ZYM system revealed that strains GGG-R5^T^ and M4-18^T^ to exhibit similar profiles as their nearest neighbors except for *β*-glucuronidase, *α*-mannosidase and *α*-fucosidase activities (Table S2). Other API based analyses indicated that strains GGG-R5^T^ and M4-18^T^ distinctly differed from their neighbors (Tables 1, S3, and S4). On comparison of strain GGG-R5^T^ with its neighbors, *M. agri* KACC 21228^T^, *M. jinjuensis* KACC 16571^T^, and *M. polytrichastri* KACC 19030^T^, differences were observed in the assimilation of D-glucose, D-maltose, D-melbiose, D-saccharose, glycogen, L-arabinose, N-acetylglucosamine, salicin, and acid production pattern amygdalin, D- and L-arabinose, D-fructose, D-galactose, D-saccharose, glycogen, L-fucose, and N-acetylglucosamine (Table S4). Several of the traits were more consistent with *M. agri* KACC 21228^T^ with which it had the most similarity. In case of strain M4-18^T^, and its nearest neighbors *M. ginsenosidivorax* KACC 14955^T^, *M. pineti* KACC 23032^T^, and *M. dorajii* KACC 14556^T^, similar pattern of carbohydrate assimilation and acid production was observed except for assimilation of D-maltose and glycogen as well as acid production from amidon, amygdalin, arbutin, D-arabinose, D-melibiose, D-melezitose, inulin, and N-acetylglucosamine (Tables 1, S3, and S4).

### Chemotaxonomic characterization

Studying the cellular fatty acids indicated that the major fatty acids were iso-C_15:0_ (28.9%, 24.7%), iso-C_17:0_ 3-OH (9.3%, 7.5%) and summed feature 3 (C_16:1_ *ω7c* and/or C_16:1_ *ω6c*) (32.5%, 37.8%), respectively for strains GGG-R5^T^ and M4-18^T^ (Table S5). Analysis of polar lipids indicated that strain GGG-R5^T^ and M4-18^T^ contained PE (phosphatidylethanolamine), phospholipids (PL) aminophospholipids (APL), and several unidentified lipids (L) (Fig. S2) consistent with members of the genus *Mucilaginibacter*. In both GGG-R5^T^ and M4-18^T^, MK-7 was the major respiratory quinone. All of these characteristics are consistent with the patterns observed in the members of the genus *Mucilaginibacter* (Aydogan et al., 2016, Kang et al., 2013; Kim et al., 2022; Pankratov et al., 2007; Zhang et al., 2019).

### Genome based phylogeny and characterization

**Fig. 2.**
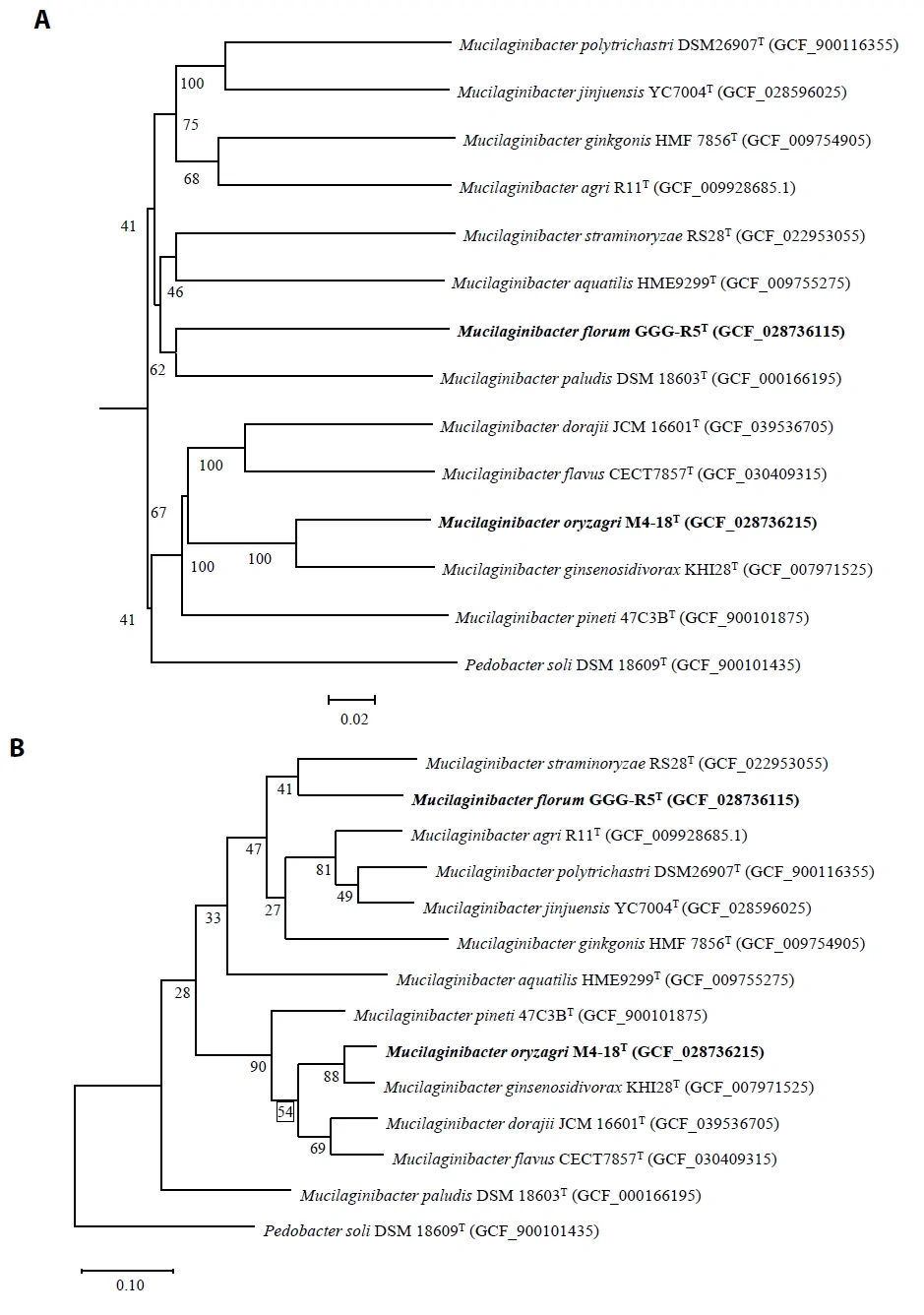
(A) Phylogenomic tree for strains M4-18^T^ and GGG-R5^T^ inferred with FastME 2.1.6.1 from GBDP (genome blast distance phylogeny) distances calculated from genome sequences. The branch lengths are scaled in terms of GBDP distance formula d_5_. The numbers above branches are GBDP pseudo-bootstrap support values from 100 replications, with an average branch support of 71.4% Genome sequence of *Pedobacter soli* DSM18609^T^ was used as outgroup. (B) Genome-based phylogenetic tree reconstructed using an up-to-date bacterial core gene (UBCG) set. Numerical values at the nodes indicate the gene support index (GSI), which is the number of single-gene trees supporting the branch. Genome sequence of *Pedobacter soli* DSM18609^T^ was used as outgroup. Scale bar, 0.10 indicates accumulated changes per nucleotide.

Genome analysis indicated that the genome size of strains GGG-R5^T^ and M4-18^T^ were at 4.8 Mb and 7.5 Mb, respectively, similar to the range of their compared neighbor strains (Tables 2 and S6). Two independent approaches, overall genome relatedness index (OGRI) studied using orthoANI as well as GGDC using the dDDH used to compare similarity at genome level indicated that the two strains could be classified as novel as values fell well below the established thresholds levels (< 95–96% for orthoANI; < 70% for dDDH) (Table 2). Genome based trees constructed using GGDC calculation showed that strains GGG-R5^T^ and M4-18^T^ formed separate clades within the genus *Mucilaginibacter* (Fig. 2). Comparison of the two genomes indicated that strain GGG-R5^T^ was smaller among the two with 4,158–4,353 coding sequences (CDS) and strain M4-18^T^ harbored 6,195–6,823 CDS (Table S7). Annotation of the genomes using RAST resulted in subsystem coverage of 20% (851 hits) and 15% (985 hits), respectively for GGG-R5^T^ and M4-18^T^ (Table S8). In both strains, carbohydrate, amino acid and protein metabolism related genes made up to the most of the highly predicted hits. When eggNOG mapper based functional annotation was carried out, a relatively higher number of hits viz. 3,623 and 5,230 were observed respectively for strains GGG-R5^T^ and M4-18^T^ (Table S9). From all these data, M4-18^T^ can be described to possess a larger genome (7.5 Mb) with a high number of genes involved in fatty acid and lipid metabolism, isoprenoid synthesis, quorum sensing, cell signaling, and plant cell wall degrading enzymes. These genes may play a significant role in colonization of plant tissues by M4-18^T^. On the other hand, genome of GGG-R5^T^ showed distinctly higher number of genes involved in virulence, iron acquisition & metabolism, as well as antibiotic resistance (Tables S8, S10, S11, S12, and S13). The COG categorization predicted for the genomes of strains GGG-R5^T^ and M4-18^T^ are given in Table S10. Search for putative secondary metabolite genes resulted in identification of carotenoid and flexirubin production loci in the genomes of strains GGG-R5^T^ and M4-18^T^ (Table S11). Mining of the genomes for antibiotic resistant loci using RGI tool indicated presence of resistance loci to fluoroquinolone and tetracycline antibiotic in both genomes (Table S12). We also found several plant growth promoting and plant-bacterial interaction factors to be present in the genome of both strains (Table S13). Gene-wise search indicated that both GGG-R5^T^ and M4-18^T^ harbored several genes earlier reported for plant growth promotional activities (Almirón et al., 2025; Kumar et al., 2022). These include genes for phytohormone synthesis namely ^trp^ABCDEF (for production of auxin indole-3-acetic acid) and *log*, *mia*AB, *yvd*D (for cytokinins). Genes for other plant growth promotional activities such as phosphate solubilization (*phn*ABOPY) and siderophore mediated iron acquisition (*ent*S, *feo*AB, and *pch*R) were also identified (Table S14) in both strains. However, we could not detect any genes responsible for motility (flagellar genes) and chemotaxis which was consistent with our earlier COG based segregation of gene function. A detailed list of genes responsible for other characteristics including colonization of plants, competitive survival as well as control of stress conditions, heavy metal detoxification and xenobiotics degradation are listed in Table S14, which indicate their potential function as beneficial bacteria which may aid plant growth. Subsequent experimental studies to confirm these characteristics might validate their potential as plant growth promoting bacteria.

### Proposal of a novel species

Phylogenetic analyses based on both 16S rRNA gene and whole-genome sequencing indicate that strains M4-18^T^ and GGG-R5^T^ fall under the genus *Mucilaginibacter*. The taxonomic position of both strains into the genus *Mucilaginibacter* was also supported by the morphological and chemotaxonomic characteristics. The strains were consistent with other members of the genus *Mucilaginibacter* in several characteristics including being Gram-stain-negative, aerobic, non-spore-forming, non-motile with production of pigment flexirubin. These characteristics are consistent with the type species for the genus *Mucilaginibacter paludis*, which included Gram-stain-negative staining, non-motile shape, sensitivity to NaCl, presence of iso-C_15:0_, iso-C_17:0_ 3-OH, C_16:1_ *ω7c* as a part of the summed feature 3 as the major fatty acids and MK-7 as the major menaquinone (Pankratov et al., 2007). However, they also differed in few characteristics from the neighboring type species in terms of enzyme profiles, assimilation of sugars and acid production substrates. The OrthoANI, dDDH, genome size and G + C content analyses for genome comparison and phenotypic differences confirmed that strains GGG-R5^T^ and M4-18^T^ were distinguishable from each other as well as from other members of the genus *Mucilaginibacter*. Based on all of the lines of evidence described above, strains GGG-R5^T^ and M4-18^T^ represent two novel species of the genus *Mucilaginibacter*, for which the names *Mucilaginibacter florum* sp. nov. and *Mucilaginibacter oryzagri* sp. nov. are proposed accordingly.

### Description of *Mucilaginibacter florum* sp. nov.

*Mucilaginibacter florum* (flo’rum. L. gen. pl. n. *florum*, of flowers).

Gram-stain-negative, non-motile and rod-shaped cells (0.6–0.65 × 1.2–2.5 μm) after 2 days on R2A medium. Colonies light-pink, convex, round and watery, and measured at 2–3 mm after 2 days of incubation at 28°C in R2A agar. Growth occurs at 4–37°C (no growth at 40°C, optimum 28°C) and pH 5.0–9.0 (optimum pH 7.0–8.0), and in the presence of 0–1% (w/v) NaCl (no growth at 2%, optimum 0%). Catalase- and oxidase-positive. Can hydrolyze casein and produce flexirubin-type pigments. Based on API 20NE test, the species tested positive for aesculin & gelatin hydrolysis, *β*-galactosidase activity but negative for nitrate reduction, indole production, glucose fermentation, arginine dihydrolase & urease activities. The API 20NE and API 32GN tests indicated positive for assimilation of D-glucose, D-maltose, D-mannose, D-mannitol, D-melibiose, D-saccharose, L-arabinose, glycogen, salicin, and N-acetylglucosamine but negative reaction for assimilation of sugars such as D-ribose, L-fucose, and L-rhamnose; sugar alcohols such as D-mannitol, D-sorbitol, and inositol; amino acids such as L-alanine, L-serine, L-proline; organic acids such as adipic acid, capric acid, hydroxybenzoic acid, itaconic acid, lactic acid, malic acid, phenylacetic acid, propionic acid, suberic acid, valeric acid, potassium gluconate, sodium malonae, sodium acetate, trisodium citrate, and 3-hydroxybutyric acid. Enzyme production studied using API ZYM test indicated that the species can produce acid & alkaline phosphatase, esterase, esterase lipase, cystine, leucine & valine arylamidases, naphthol-AS-BI-phosphohydrolase, *α* and *β*-galactosidases, *α* and *β*-glucosidases, *β*-glucuronidase, and N-acetyl-*β*-glucosaminidase but cannot produce lipase, trypsin, *α*-chymotrypsin, *α*-fucosidase, and *α*-mannosidase. The major cellular fatty acids are iso-C_15:0_, iso-C_17:0 3-OH_ and summed feature 3 (C_16:1_ *ω7c* and/or C_16:1_ *ω6c*). The major menaquinone is MK-7, and polar lipids composed of PE, PL, APL, and several unidentified lipids.

The type strain is GGG-R5^T^ (= KACC 22063^T^ = JCM 36590^T^) (GenBank accession PP236896 & CP117877), isolated from flowers of golden wave (*Coreopsis grandiflora*), Jeonju, from the Republic of Korea. The G + C content of the type strain based on WGS is 41.7%.

### Description of *Mucilaginibacter oryzagri* sp. nov.

*Mucilaginibacter oryzagri* (o.ryz.a’gri. L. fem. n. *oryza*, rice; L. masc. n. *ager*, a field; N.L. gen. n. *oryzagri*, of a rice field).

Gram-stain-negative, non-motile, rod-shaped cells (0.6–0.7 × 2.0–8.0 μm) after 2 days on R2A medium. Colonies milky with a yellow tinge and irregular with a diameter smaller than 0.5 mm after 2 days of incubation in R2A agar. Growth occurs at 4–35°C (no growth at 37°C, optimum 28°C) and pH 5.0–8.0 (optimum pH 7.0), and at 0–1% (w/v) NaCl (no growth at 2%, optimum 0%). Catalase- and oxidase-positive. Flexirubin-type pigments are produced but casein not hydrolyzed. Based on API 20NE test, the species tested positive for aesculin & gelatin hydrolysis, *β*-galactosidase activity but negative for nitrate reduction, indole production, glucose fermentation, arginine dihydrolase & urease activities. The API 20NE and API 32GN tests indicated positive for assimilation of D-glucose, D-maltose, D-mannose, D-mannitol, D-saccharose, and L-arabinose but negative reaction for assimilation of sugars such as D-melibiose, D-ribose, L-fucose, and L-rhamnose; sugar alcohols such as D-mannitol, D-sorbitol, and inositol; amino acids such as L-alanine, L-serine, and L-proline; organic acids such as adipic acid, capric acid, hydroxybenzoic acid, itaconic acid, lactic acid, malic acid, phenylacetic acid, propionic acid, suberic acid, valeric acid, potassium gluconate, sodium malonae, sodium acetate, and trisodium citrate 3-hydroxybutyric; other substrates including glycogen, N-acetylglucosamine, and salicin. Enzyme production studied using API ZYM test indicated that the species can produce acid & alkaline phosphatase, esterase, esterase lipase, cystine, leucine & valine arylamidases, naphthol-AS-BI-phosphohydrolase, *α* and *β*-galactosidases, *α* and *β*-glucosidases, *α*-mannosidase and N-acetyl-*β*-glucosaminidase but cannot produce lipase, trypsin, *α*-chymotrypsin, *α*-fucosidase, and *β*-glucuronidase. The major menaquinone is MK-7, and the major cellular fatty acids were iso-C_15:0_, iso-C_17:0 3-OH_ and summed feature 3 (C_16:1_ *ω7c* and/or C_16:1_ *ω6c*). Polar lipids composed of PE, PL, APL, and several unidentified lipids.

The type strain is M4-18^T^ (=KACC 22773^T^ =JCM 35894^T^ (GenBank accession OP377360 & CP117883), isolated from soil from Miryang, Republic of Korea. The G + C content of the type strain based on WGS is 43.1%.

## Figures and Tables

**Table 1.** Differential characteristics of strains GGG–R5^T^ and M4–18^T^ and their closest phylogenetic neighbors Strains: 1, GGG–R5^T^; 2, M4–18^T^; 3, *Mucilaginibacter agri* KACC 21228^T^; 4, *Mucilaginibacter jinjuensis* KACC 16571^T^; 5, *Mucilaginibacter polytrichastri* KACC 19030^T^; 6, *Mucilaginibacter straminoryzae* KACC 24163^T^; 7, *Mucilaginibacter ginsenosidivorax* KACC 14955^T^; 8, *Mucilaginibacter pineti* KACC 23032^T^; 9, *Mucilaginibacter flavus* KACC 18090^T^; 10, *Mucilaginibacter dorajii* KACC 14556^T^; 11, *Mucilaginibacter paludis* TPT56^T^.

Characteristics	1	2	3	4	5	6	7	8	9	10	11
Isolation source	Flower	Soil	Soil^a^	Rotten wood^b^	Moss^c^	Rice straw^d^	Sediment^e^	Pine tree^f^	Wetland^g^ water	Plant rhizosphere^h^	Peat bog^i^
Cell size (µm)	0.6–0.65 × 1.2–2.5	0.6–0.7 × 2.0–8.0	0.2–0.4 × 1.4–1.8^a^	0.35–0.4 × 1.2–1.4^b^	0.6–0.7 × 2.0–3.2^c^	0.4–0.5 × 1.5–1.8^d^	0.4–0.6 × 1.3–2.4^e^	0.09–0.1 × 1–3^f^	NA	0.6–0.8 × 1.1–1.8^h^	0.5–0.8^i^
Colony color	Light–pink	Milky	Orange^a^	Pale–orange^b^	Orange^c^	Light pink^d^	Pale pink^e^	Light pink^f^	Pale yellow^g^	Light yellow^h^	light pink to reddish^i^
Temperature range (optimum, °C)	4–37 (28)	4–35 (28)	25–37 (30)^a^	4–35 (30)^b^	4–30	17–40	10–42	15–30	10–30	4–30	20–25^i^
					(28–30)^c^	(25)^d^	(30–37)^e^	(26)^f^	(30)^g^	(20–25)^h^	
Salinity range (optimum, %, w/v)	0–1 (0)	0–1 (0)	0–0.5 (0)^a^	0–1 (0)^b^	0–3 (0)^c^	0–2 (0)^d^	0^e^	0–0.3^f^	0–0.5	0–1 (0)^h^	0–1 (0)^i^
									(0.5)^g^		
pH range (optimum)	5.0–9.0	5.0–8.0	5.0–8.0	4.0–8.5	5.0–8.0	5.5–8.0 (7.0)^d^	5.5–8.5^e^	5.5–8.0	5.0–9.0	5.0–8.0	6.0–6.5^i^
	(7–8)	(7)	(7.5)^a^	(6.0)^b^	(6.5–7.0)^c^			(7.0)^f^	(7.0)^g^	(5.5–6.0)^h^	
Nitrate reduction	–/–	–/–	–/–	–/–	–/–	–/–	–/–	–/–	–/–	–/–	NA
Gelatin hydrolysis	+	+	–	–	–	–	+	–	–	–	NA
**Enzyme activity**											
Catalase	+	+	+	+	*–*	+	+	+	+	+	+^i^
Oxidase	+	+	+	+	+	+	+	+	+	+	+^i^
*β*–glucuronidase	+	–	–	–	–	+	–	–	–	–	NA
*α*–mannosidase	–	+	+	+	+	+	+	+	+	+	NA
*α–*fucosidase	–	–	–	–	–	+	+	+	+	+	NA
**Assimilation of:**											
*N*–acetylglucosamine	+	–	+	+	–	+	–	+	–	–	+^i^
Glycogen	+	–	+	–	–	–	–	+	–	+	NA
Salicin	+	–	+	–	–	+	–		–	–	–^i^
D–melibiose	+	–	+	+	–	+	–	+	–	–	–^i^
**Acid production from:**											
D–galactose	+	+	–	–	–	+	+	+	+	+	+^i^
D–fructose	+	+	–	–	–	+	+	+	–	+	+^i^
Inulin	–	+	–	–	–	–	–	+	–	–	–^i^

a Seo et al., 2020;

b Khan et al., 2013b;

c Chen et al., 2014;

d Ko et al., 2023;

e Kim et al., 2013;

f Paiva et al., 2014;

g Joung et al., 2014;

h Kim et al., 2010;

i Pankratov et al., 2007.

NA, data not available.

**Table 2.** Genome based comparison-using OrthoANI, dDDH and G + C content analyses of strains GGG-R5^T^ and M4-18^T^ and related type strains

Strain	Type strains	Total length (bp)	OrthoANI value (%)	dDDH (%)	G + C content (%)
GGG-R5^T^		4,809,148	-	-	41.7
GGG-R5^T^ vs	*M. paludis* TPT56^T^	8,408,322	75.2	21.3	42.9
	*M. agri* R11^T^	5,598,559	73.5	19.4	42.7
	*M. jinjuensis* KACC 16571^T^	6,157,393	74.0	19.5	42.1
	*M. polytrichastri* DSM 26907^T^	5,813,139	73.9	19.1	42.0
	*M. straminoryzae* RS28^T^	4,754,355	73.3	19.7	44.7
M4-18^T^		7,541,737	-	-	43.1
M4-18^T^ vs	*M. paludis* TPT56^T^	8,408,322	74.6	21.0	42.9
	*M. ginsenosidivora×* KHI28^T^	7,811,413	88.5	36.6	43.1
	*M. pineti* 47C3B^T^	7,200,555	76.7	21.9	42.9
	*M. flavus* CECT7857^T^	6,948,697	78.6	22.4	42.4
	*M. dorajii* JCM 16601^T^	7,153,246	78.9	23.0	42.6

## Data Availability

All results generated in this study are included in the article and supplementary information. Sequence data from this study are available from NCBI (https://www.ncbi.nlm.nih.gov/) and can be accessed using the accession numbers provided in “Nucleotide Sequence Accession Numbers” section of this article.

## Supplementary Information

Table S1. Comparison of results of API 20 NE tests for strains GGG–R5^T^ and M4–18^T^ with their nearest phylogenetic neighbors. Strains: 1, GGG–R5^T^; 2, M4–18^T^; 3, *Mucilaginibacter agri* KACC 21228^T^; 4, *Mucilaginibacter jinjuensis* KACC 16571^T^; 5, *Mucilaginibacter polytrichastri* KACC 19030^T^; 6, *Mucilaginibacter straminoryzae* KACC 24163^T^; 7, *Mucilaginibacter ginsenosidivorax* KACC 14955^T^; 8, *Mucilaginibacter pineti* KACC 23032^T^; 9, *Mucilaginibacter flavus* KACC 18090^T^; 10, *Mucilaginibacter dorajii* KACC 14556^T^; 11, *Mucilaginibacter paludis* TPT56^T^. +, positive; –, negative; W, weak

Table S2. Comparison of enzyme activities of strains GGG–R5^T^ and M4–18^T^ with their nearest phylogenetic neighbors. Strains: 1, GGG–R5^T^; 2, M4–18^T^; 3, *Mucilaginibacter agri* KACC 21228^T^; 4, *Mucilaginibacter jinjuensis* KACC 16571^T^; 5, *Mucilaginibacter polytrichastri* KACC 19030^T^; 6, *Mucilaginibacter straminoryzae* KACC 24163^T^; 7, *Mucilaginibacter ginsenosidivorax* KACC 14955^T^; 8, *Mucilaginibacter pineti* KACC 23032^T^; 9, *Mucilaginibacter flavus* KACC 18090^T^; 10, *Mucilaginibacter dorajii* KACC 14556^T^; 11, *Mucilaginibacter paludis* TPT56^T^. +, positive; –, negative; W, weak

Table S3. Comparison of various substrate assimilation by strains GGG–R5^T^ and M4–18^T^ with their nearest phylogenetic neighbors. Strains: 1, GGG–R5^T^; 2, M4–18^T^; 3, *Mucilaginibacter agri* KACC 21228^T^; 4, *Mucilaginibacter jinjuensis* KACC 16571^T^; 5, *Mucilaginibacter polytrichastri* KACC 19030^T^; 6, *Mucilaginibacter straminoryzae* KACC 24163^T^; 7, *Mucilaginibacter ginsenosidivorax* KACC 14955^T^; 8, *Mucilaginibacter pineti* KACC 23032^T^; 9, *Mucilaginibacter flavus* KACC 18090^T^; 10, *Mucilaginibacter dorajii* KACC 14556^T^; 11, *Mucilaginibacter paludis* TPT56^T^. +, positive; –, negative; W, weak

Table S4. Comparison of acid production from carbon sources among strains GGG–R5^T^ and M4–18^T^, and their nearest phylogenetic neighbors. Strains: 1, GGG–R5^T^; 2, M4–18^T^; 3, *Mucilaginibacter agri* KACC 21228^T^; 4, *Mucilaginibacter jinjuensis* KACC 16571^T^; 5, *Mucilaginibacter polytrichastri* KACC 19030^T^; 6, *Mucilaginibacter straminoryzae* KACC 24163^T^; 7, *Mucilaginibacter ginsenosidivorax* KACC 14955^T^; 8, *Mucilaginibacter pineti* KACC 23032^T^; 9, *Mucilaginibacter flavus* KACC 18090^T^; 10, *Mucilaginibacter dorajii* KACC 14556^T^; 11, *Mucilaginibacter paludis* TPT56^T^. +, positive; –, negative; W, weak

Table S5. Cellular fatty acid profiles of strains GGG–R5^T^ and M4–18^T^, and related type strains. Strains: 1, GGG–R5^T^; 2, M4–18^T^; 3, *Mucilaginibacter agri* KACC 21228^T^; 4, *Mucilaginibacter jinjuensis* KACC 16571^T^; 5, *Mucilaginibacter polytrichastri* KACC 19030^T^; 6, *Mucilaginibacter straminoryzae* KACC 24163^T^; 7, *Mucilaginibacter ginsenosidivorax* KACC 14955^T^; 8, *Mucilaginibacter pineti* KACC 23032^T^; 9, *Mucilaginibacter flavus* KACC 18090^T^; 10, *Mucilaginibacter dorajii* KACC 14556^T^; 11, *Mucilaginibacter paludis* TPT56^T^. Values more than 10% of the total fatty acids were bolded. –, not detected or < 1.0% of the total fatty acids.

Table S6. Comparison of genome characteristics of strains GGG–R5^T^ and M4–18^T^ along with their nearest phylogenetic neighbors NCBI–Genome database

Table S7. Comparison of annotation statistics when using different tools to analyze genomes of strains GGG–R5^T^ and M4–18^T^.

Table S8. Functional annotation of the genome of strains GGG–R5^T^ and M4–18^T^ using RAST

Table S9. Functional annotation of the genome of strains GGG–R5^T^ and M4–18^T^ using RAST and eggNOG mapper

Table S10. Clusters of Orthologous Genes (COGs) predicted for genomes of strains GGG–R5^T^ and M4–18^T^

Table S11. Secondary metabolites predicted using antiSMASH bacterial version 7.0

Table S12. Resistance loci to antibiotics detected using the Resistance Gene Identifier (RGI) tool

Table S13. Plant bacterial only interaction factors and plant growth promoting traits predicted using PLaBAse

Table S14. List of putative functional plant-growth promoting genes predicted in strains M4-18T and GGG-R5T using PLaBAse-PGPT-Pred

Fig. S1. Transmission electron micrographs of strains GGG–R5^T^ (A–C) and M4–18^T^ (D–F). Bar, 2 μm.

Fig. S2. Polar lipid profiles of strains GGG–R5^T^ (A) and M4–18^T^ (B) after spraying 5% ethanolic phosphomolybdic acid (for total lipids); ninhydrin (Sigma) (for aminolipids); molybdenum blue (Sigma) (for phospholipids); α–naphtol – sulphuric acid (for glycolipids); Dragendorff reagent (for Choline–containing lipids). PDE, phosphatidyldimethylethanolamine; PE, phosphatidylethanolamine; PL, unidentified phospholipid; APL, unidentified aminophospholipids; AL, unidentified aminolipids; L, unidentified lipid.
