# Supplementary material for: Mucilaginibacter florum sp. nov., isolated from the flower of Coreopsis grandiflora and Mucilaginibacter oryzagri sp. nov., isolated from rice paddy soil in Korea
Source: J Microbiol. 2025 Dec 31;63(12):e2509014. doi: 10.71150/jm.2509014 (PMC13577788; doi:10.71150/jm.2509014)
Supplement: Supplementary file 1 [file jm-2509014-Supplementary-Table-S1.pdf]

Table S1. Comparison of results of API 20 NE tests for strains GGG–R5^{T} and M4–18^{T} with their nearest phylogenetic neighbors.

Strains: 1, GGG–R5^{T}; 2, M4–18^{T}; 3, *Mucilaginibacter agri* KACC 21228^{T}; 4, *Mucilaginibacter jinjuensis* KACC 16571^{T}; 5, *Mucilaginibacter polytrichastri* KACC 19030^{T}; 6, *Mucilaginibacter straminoryzae* KACC 24163^{T}; 7, *Mucilaginibacter ginsenosidivorax* KACC 14955^{T}; 8, *Mucilaginibacter pineti* KACC 23032^{T}; 9, *Mucilaginibacter flavus* KACC 18090^{T}; 10, *Mucilaginibacter dorajii* KACC 14556^{T}; 11, *Mucilaginibacter paludis* TPT56^{T}. +, positive; –, negative; W, weak

**Characteristics**	**1**	**2**	**3**	**4**	**5**	**6**	**7**	**8**	**9**	**10**	**11**[a]
Nitrate reduction	–/–	–/–	–/–	–/–	–/–	–/–	–/–	–/–	–/–	–/–	NA
Indole production	–	–	–	–	–	–	–	–	–	–	–
Glucose fermentation	–	–	–	–	–	W	–	–	–	–	+
Arginine dihydrolase	–	–	–	–	–	–	–	–	–	–	NA
Urease	–	–	–	–	–	–	–	–	–	–	NA
Aesculin hydrolysis	+	+	+	+	+	+	+	+	+	+	–
Gelatin hydrolysis	+	+	–	–	–	–	+	–	–	–	–
β-galactosidase	+	+	+	+	+	+	+	+	+	+	–
**Assimilation:**											
D-glucose	+	+	+	+	–	+	–	+	+	+	+
L-arabinose	+	+	+	–	–	+	–	+	+	+	NA
D-mannose	+	–	+	–	–	+	–	–	+	+	+
D-mannitol	–	–	–	–	–	–	–	–	–	+	–
N-acetylglucosamine	+	–	+	–	–	+	–	+	–	–	+
D-maltose	+	+	+	–	–	+	–	+	–	–	+
Potassium gluconate	–	–	–	–	–	–	–	–	–	–	NA
Capric acid	–	–	–	–	–	–	–	–	–	–	NA
Adipic acid	–	–	–	–	–	–	–	–	–	–	NA
Malic acid	–	–	–	–	–	–	–	–	–	–	–
Trisodium citrate	–	–	–	–	–	–	–	–	–	–	–
Phenylacetic acid	–	–	–	–	–	–	–	–	–	–	NA

[a]Pankratov et al., 2007
