# Supplementary material for: Mucilaginibacter florum sp. nov., isolated from the flower of Coreopsis grandiflora and Mucilaginibacter oryzagri sp. nov., isolated from rice paddy soil in Korea
Source: J Microbiol. 2025 Dec 31;63(12):e2509014. doi: 10.71150/jm.2509014 (PMC13577788; doi:10.71150/jm.2509014)
Supplement: Supplementary file 2 [file jm-2509014-Supplementary-Table-S10.pdf]

Table S10. Clusters of Orthologous Genes (COGs) predicted for genomes of strains GGG–R5^T and M4–18^T

**COG CATAGORIES**	***Mucilaginibacter* sp. GGG–R5^T**	***Mucilaginibacter* sp. M4–18^T**
INFORMATION STORAGE AND PROCESSING	**534**	**770**
[J] Translation, ribosomal structure and biogenesis	159	180
[A] RNA processing and modification	0	0
[K] Transcription	244	399
[L] Replication, recombination and repair	129	189
[B] Chromatin structure and dynamics	2	2
CELLULAR PROCESSES AND SIGNALING	**721**	**1023**
[D] Cell cycle control, cell division, chromosome partitioning	31	41
[Y] Nuclear structure	0	0
[V] Defense mechanisms	92	120
[T] Signal transduction mechanisms	167	262
[M] Cell wall/membrane/envelope biogenesis	305	378
[N] Cell motility	15	26
[Z] Cytoskeleton	0	0
[W] Extracellular structures	0	0
[U] Intracellular trafficking, secretion, and vesicular transport	0	56
[O] Posttranslational modification, protein turnover, chaperones	111	140
METABOLISM	**1263**	**1835**
[C] Energy production and conversion	197	256
[G] Carbohydrate transport and metabolism	247	450
[E] Amino acid transport and metabolism	240	319
[F] Nucleotide transport and metabolism	74	90
[H] Coenzyme transport and metabolism	127	159
[I] Lipid transport and metabolism	152	213
[P] Inorganic ion transport and metabolism	171	276
[Q] Secondary metabolites biosynthesis, transport and catabolism	55	72
POORLY CHARACTERIZED	**791**	**1202**
[R] General function prediction only	0	0
[S] Function unknown	791	1202
No hits	282	400
**TOTAL**	3591	5230
