# Supplementary material for: Mucilaginibacter florum sp. nov., isolated from the flower of Coreopsis grandiflora and Mucilaginibacter oryzagri sp. nov., isolated from rice paddy soil in Korea
Source: J Microbiol. 2025 Dec 31;63(12):e2509014. doi: 10.71150/jm.2509014 (PMC13577788; doi:10.71150/jm.2509014)
Supplement: Supplementary file 3 [file jm-2509014-Supplementary-Table-S11.pdf]

Table S11. Secondary metabolites predicted using antiSMASH bacterial version 7.0

Region	Type	From	To	Most similar known cluster	Similarity
**Mucilaginibacter sp. GGG–R5^{T} (CP117877)**					
Region 1	NI–siderophore	332,048	361,814		
Region 2	lanthipeptide–class–i	669,423	699,534		
Region 3	terpene	1,286,204	1,307,037	carotenoid - Terpene	42%
Region 4	pyrrolidine	1,591,921	1,615,302		
Region 5	T1PKS,NRPS–like	2,105,899	2,152,954		
Region 6	NRPS,T1PKS	2,188,452	2,255,353		
Region 7	crocagin	3,077,811	3,101,402		
Region 8	NAPAA	3,101,406	3,135,374		
Region 9	resorcinol,arylpolyene,T3PKS	4,317,909	4,376,877	flexirubin - Polyketide	22%
Region 10	lanthipeptide–class–i	4,577,478	4,608,952		
**Mucilaginibacter sp. M4–18^{T} (CP117883)**					
Region 1	T3PKS	87,846	128,958		
Region 2	lanthipeptide–class–iv	675,346	697,964		
Region 3	RiPP–like	2,274,804	2,285,724		
Region 4	RRE–containing	2,654,663	2,674,911		
Region 5	terpene	4,199,607	4,220,443	carotenoid - Terpene	28%
Region 6	lanthipeptide–class–i	6,706,936	6,731,227		
Region 7	arylpolyene,resorcinol	6,987,059	7,056,328	Flexirubin-Other	72%
