# Supplementary material for: Mucilaginibacter florum sp. nov., isolated from the flower of Coreopsis grandiflora and Mucilaginibacter oryzagri sp. nov., isolated from rice paddy soil in Korea
Source: J Microbiol. 2025 Dec 31;63(12):e2509014. doi: 10.71150/jm.2509014 (PMC13577788; doi:10.71150/jm.2509014)
Supplement: Supplementary file 4 [file jm-2509014-Supplementary-Table-S12.pdf]

Table S12. Resistance loci to antibiotics detected using the Resistance Gene Identifier (RGI) tool

RGI Criteria	ARO Term	SNP	Detection Criteria	AMR Gene Family	Drug Class	Resistance Mechanism	% Identity of Matching Region	% Length of Reference Sequence
**Mucilaginibacter sp. GGG–R5 (CP117877)**								
Strict	adeF		protein homolog model	resistance–nodulation–cell division (RND) antibiotic efflux pump	fluoroquinolone antibiotic, tetracycline antibiotic	antibiotic efflux	41.8	99.7
Strict	adeF		protein homolog model	resistance–nodulation–cell division (RND) antibiotic efflux pump	fluoroquinolone antibiotic, tetracycline antibiotic	antibiotic efflux	40.5	100
Strict	vanT gene in vanG cluster		protein homolog model	glycopeptide resistance gene cluster, vanT	glycopeptide antibiotic	antibiotic target alteration	31.1	100
Strict	adeF		protein homolog model	resistance–nodulation–cell division (RND) antibiotic efflux pump	fluoroquinolone antibiotic, tetracycline antibiotic	antibiotic efflux	42.1	100
**Mucilaginibacter sp. M4–18 (CP117883)**								
Strict	adeF		protein homolog model	resistance–nodulation–cell division (RND) antibiotic efflux pump	fluoroquinolone antibiotic, tetracycline antibiotic	antibiotic efflux	42.9	100
