# Supplementary material for: Mucilaginibacter florum sp. nov., isolated from the flower of Coreopsis grandiflora and Mucilaginibacter oryzagri sp. nov., isolated from rice paddy soil in Korea
Source: J Microbiol. 2025 Dec 31;63(12):e2509014. doi: 10.71150/jm.2509014 (PMC13577788; doi:10.71150/jm.2509014)
Supplement: Supplementary file 5 [file jm-2509014-Supplementary-Table-S13.pdf]

Table S13. Plant bacterial only interaction factors and plant growth promoting traits predicted using PLaBAse

**Prediction tools**	**Mucilaginibacter sp. GGG–R5**	**Mucilaginibacter sp. M4–18**
**PIFAR**		
Adhesion	20	46
Detoxification	38	45
EPS	138	154
Hormone	37	36
LPS	10	9
MDRs	42	41
Metabolism	13	16
Movement	5	10
PCWDE (plant cell wall degrading enzymes)	8	29
Pigment	11	13
Protease	3	8
Toxin	88	115
Volatiles	4	2
	417	524
**PGPT**		
Bio–fertilization	480	658
Bio–remediation	307	452
Phytohormone/ plant signal production	315	398
Colonizing plant system	975	1500
Competitive exclusion	943	1359
Plant immune response stimulation	57	62
Stress control/ biocontrol	758	1032
Putative functions	13	23
	3848	5484
