# Supplementary material for: Mucilaginibacter florum sp. nov., isolated from the flower of Coreopsis grandiflora and Mucilaginibacter oryzagri sp. nov., isolated from rice paddy soil in Korea
Source: J Microbiol. 2025 Dec 31;63(12):e2509014. doi: 10.71150/jm.2509014 (PMC13577788; doi:10.71150/jm.2509014)
Supplement: Supplementary file 6 [file jm-2509014-Supplementary-Table-S2.pdf]

Table S2. Comparison of enzyme activities of strains GGG–R5^T and M4–18^T with their nearest phylogenetic neighbors. Strains: 1, GGG–R5^T; 2, M4–18^T; 3, *Mucilaginibacter agri* KACC 21228^T; 4, *Mucilaginibacter jinjuensis* KACC 16571^T; 5, *Mucilaginibacter polytrichastri* KACC 19030^T; 6, *Mucilaginibacter straminoryzae* KACC 24163^T; 7, *Mucilaginibacter ginsenosidivorax* KACC 14955^T; 8, *Mucilaginibacter pineti* KACC 23032^T; 9, *Mucilaginibacter flavus* KACC 18090^T; 10, *Mucilaginibacter dorajii* KACC 14556^T; 11, *Mucilaginibacter paludis* TPT56^T. +, positive; –, negative; W, weak

**Characteristics**	**1**	**2**	**3**	**4**	**5**	**6**	**7**	**8**	**9**	**10**	**11**[a]
Alkaline phosphatase	+	+	+	+	+	+	+	+	+	+	NA
Esterase (C4)	+	+	+	+	+	+	+	+	+	+	NA
Esterase lipase (C8)	+	+	+	+	+	+	+	–	+	+	NA
Lipase (C14)	–	–	W	–	W	–	–	–	–	–	NA
Leucine arylamidase	+	+	+	+	+	+	+	+	+	+	NA
Valine arylamidase	+	+	+	+	+	+	+	+	+	+	NA
Cystine arylamidase	+	+	+	+	+	+	+	+	+	+	NA
Trypsin	–	–	–	–	–	+	–	–	–	–	NA
α–chymotrypsin	–	–	–	–	–	–	–	–	–	–	NA
Acid phosphatase	+	+	+	+	+	+	+	+	+	+	NA
Naphthol–AS–BI–phosphohydrolase	+	+	+	+	+	+	+	+	+	+	NA
α–galactosidase	+	+	+	+	+	+	+	+	+	+	NA
β–galactosidase	+	+	+	+	+	+	+	+	+	+	NA
β–glucuronidase	+	–	–	–	–	+	–	–	–	–	NA
α–glucosidase	+	+	+	+	+	+	+	+	+	+	NA
β–glucosidase	+	+	+	+	+	+	+	+	+	+	NA
N–acetyl–*β*–glucosaminidase	+	+	+	+	+	+	+	+	+	+	NA
α–mannosidase	–	+	+	+	+	+	+	+	+	+	NA
α–fucosidase	–	–	–	–	–	+	+	+	+	+	NA

[a] (Pankratov et al., 2007)
