# Supplementary material for: Mucilaginibacter florum sp. nov., isolated from the flower of Coreopsis grandiflora and Mucilaginibacter oryzagri sp. nov., isolated from rice paddy soil in Korea
Source: J Microbiol. 2025 Dec 31;63(12):e2509014. doi: 10.71150/jm.2509014 (PMC13577788; doi:10.71150/jm.2509014)
Supplement: Supplementary file 7 [file jm-2509014-Supplementary-Table-S3.pdf]

Table S3. Comparison of various substrate assimilation by strains GGG–R5^{T} and M4–18^{T} with their nearest phylogenetic neighbors. Strains: 1, GGG–R5^{T}; 2, M4–18^{T}; 3, *Mucilaginibacter agri* KACC 21228^{T}; 4, *Mucilaginibacter jinjuensis* KACC 16571^{T}; 5, *Mucilaginibacter polytrichastri* KACC 19030^{T}; 6, *Mucilaginibacter straminoryzae* KACC 24163^{T}; 7, *Mucilaginibacter ginsenosidivorax* KACC 14955^{T}; 8, *Mucilaginibacter pineti* KACC 23032^{T}; 9, *Mucilaginibacter flavus* KACC 18090^{T}; 10, *Mucilaginibacter dorajii* KACC 14556^{T}; 11, *Mucilaginibacter paludis* TPT56^{T}.   +, positive; –, negative; W, weak

**Assimilation**	**1**	**2**	**3**	**4**	**5**	**6**	**7**	**8**	**9**	**10**	**11[a]**
L-rhamnose	–	–	–	–	–	–	–	–	–	–	+
N-acetylglucosamine	+	–	+	+	–	+	–	+	–	–	+
D-ribose	–	–	–	–	–	–	–	–	–	–	–
Inositol	–	–	–	–	–	–	–	–	–	–	+
D-saccharose	+	+	+	+	–	+	–	+	+	+	NA
D-maltose	+	+	+	+	–	+	–	+	+	–	+
Itaconic acid	–	–	–	–	–	–	–	–	–	–	NA
Suberic acid	–	–	–	–	–	–	–	–	–	–	NA
Sodium malonate	–	–	–	–	–	–	–	–	–	–	NA
Sodium acetate	–	–	–	–	–	–	–	–	–	–	–
Lactic acid	–	–	–	–	–	–	–	–	–	–	NA
L-alanine	–	–	–	–	–	–	–	–	–	–	NA
Potassium 5-ketogluconate	–	–	–	–	–	–	–	–	–	–	NA
Glycogen	+	–	+	–	–	–	–	+	–	+	NA
3-hydroxybutyric acid	–	–	–	–	–	–	–	–	–	–	–
L-serine	–	–	–	–	–	–	–	–	–	–	NA
D-mannitol	–	–	–	–	–	–	–	–	–	–	–
D-glucose	+	+	+	+	–	+	–	+	+	+	+
Salicin	+	–	+	–	–	+	–		–	–	–
D-melibiose	+	–	+	+	–	+	–	+	–	–	–
L-fucose	–	–	–	–	–	–	–	–	–	–	NA
D-sorbitol	–	–	–	–	–	–	–	–	–	–	–
L-arabinose	+	+	+	–	–	+	–	+	+	–	–
Propionic acid	–	–	–	–	–	–	–	–	–	–	NA
Capric acid	–	–	–	–	–	–	–	–	–	–	NA
Valeric acid	–	–	–	–	–	–	–	–	–	–	–
Trisodium citrate	–	–	–	–	–	–	–	–	–	–	–
L-histidine	–	–	–	–	–	–	–	–	–	–	NA
Potassium 2-ketogluconate	–	–	–	–	–	–	–	–	–	–	NA
3-hydroxybenzoic acid	–	–	–	–	–	–	–	–	–	–	NA
4-hydroxybenzoic acid	–	–	–	–	–	–	–	–	–	–	NA
L-proline	–	–	–	–	–	–	–	+	–	–	NA

[a] (Pankratov et al., 2007)
