# Supplementary material for: Mucilaginibacter florum sp. nov., isolated from the flower of Coreopsis grandiflora and Mucilaginibacter oryzagri sp. nov., isolated from rice paddy soil in Korea
Source: J Microbiol. 2025 Dec 31;63(12):e2509014. doi: 10.71150/jm.2509014 (PMC13577788; doi:10.71150/jm.2509014)
Supplement: Supplementary file 8 [file jm-2509014-Supplementary-Table-S4.pdf]

Table S4. Comparison of acid production from carbon sources among strains GGG–R5^T and M4–18^T, and their nearest phylogenetic neighbors. Strains: 1, GGG–R5^T; 2, M4–18^T; 3, *Mucilaginibacter agri* KACC 21228^T; 4, *Mucilaginibacter jinjuensis* KACC 16571^T; 5, *Mucilaginibacter polytrichastri* KACC 19030^T; 6, *Mucilaginibacter straminoryzae* KACC 24163^T; 7, *Mucilaginibacter ginsenosidivorax* KACC 14955^T; 8, *Mucilaginibacter pineti* KACC 23032^T; 9, *Mucilaginibacter flavus* KACC 18090^T; 10, *Mucilaginibacter dorajii* KACC 14556^T; 11, *Mucilaginibacter paludis* TPT56^T. +, positive; –, negative; W, weak

**Acid production from**	**1**	**2**	**3**	**4**	**5**	**6**	**7**	**8**	**9**	**10**	**11**[a]
Glycerol	–	–	–	–	–	–	–	–	–	–	NA
Erythritol	–	–	–	–	–	–	–	–	–	–	NA
D-arabinose	+	+	–	–	–	+	–	–	W	+	NA
L-arabinose	–	+	+	–	–	+	+	+	+	+	NA
D-ribose	–	–	–	–	–	–	–	–	–	–	NA
D-xylose	+	+	+	+	+	+	+	–	+	+	+
L-xylose	–	–	–	–	–	–	–	–	–	–	+
D-adonitol	–	–	–	–	–	–	–	–	–	–	NA
Methyl-β-D-xylopyranoside	–	–	–	–	–	–	–	–	–	–	NA
D-galactose	+	+	–	–	–	+	+	+	+	+	+
D-glucose	+	+	+	+	+	+	+	+	+	+	+
D-fructose	+	+	–	–	–	+	+	+	–	+	+
D-mannose	+	-	+	+	+	+	+	+	+	+	NA
L-sorbose	–	–	–	–	–	–	–	–	–	–	NA
L-rhamnose	–	–	–	–	–	–	–	–	–	–	+
Dulcitol	–	–	–	–	–	–	–	–	–	–	NA
Inositol	–	–	–	–	–	–	–	–	–	–	NA
D-mannitol	–	–	–	–	–	–	–	–	–	–	NA
D-sorbitol	–	–	–	–	–	–	–	–	–	–	NA
Methyl-α-D-mannopyranoside	–	–	–	–	–	+	–	–	–	–	NA
Methyl-α-D-glucopyranoside	–	–	–	–	–	+	–	–	–	+	NA
N-acetylglucosamine	+	+	+	+	–	+	–	+	W	+	NA
Amygdalin	–	+	–	+	–	+	–	–	–	+	NA
Arbutin	+	+	+	+	+	+	–	–	–	–	NA
Esculin ferric citrate	+	+	+	+	+	+	+	+	+	+	NA
Salicin	+	-	+	+	–	+	–	–	–	–	NA
D-cellobiose	+	+	+	+	+	+	+	+	+	+	+
D-maltose	+	+	+	+	+	+	+	+	+	+	NA
D-lactose	+	+	+	–	+	+	+	+	+	+	+
D-melibiose	+	–	+	+	+	+	+	+	+	+	–
D-saccharose	+	+	+	–	–	+	+	+	+	+	NA
D-trehalose	+	+	+	+	–	+	+	–	+	+	–
Inulin	–	+	–	–	–	–	–	+	–	–	NA
D-melezitose	–	+	–	–	–	+	+	–	–	–	NA
D-raffinose	–	+	+	–	–	+	+	+	–	+	NA
Amidon	+	+	+	+	–	+	–	–	–	+	NA
Glycogen	+	–	+	–	–	–	–	–	–	+	NA
Xylitol	–	–	–	–	–	–	–	–	–	–	NA
Gentiobiose	+	+	+	+	–	+	–	+	+	+	NA
D-turanose	–	+	–	–	–	+	+	–	+	–	NA
D-lyxose	–	–	–	–	–	–	–	–	–	–	NA
D-tagatose	–	–	–	–	–	–	–	–	–	–	NA

Acid production from	1	2	3	4	5	6	7	8	9	10	11[a]
D-fucose	–	–	–	–	–	–	–	–	–	–	NA
L-fucose	–	–	–	–	–	–	+	–	–	–	NA
D-arabitol	–	–	–	–	–	–	–	–	–	–	NA
L-arabitol	–	–	–	–	–	–	–	–	–	–	NA
Potassium gluconate	–	–	–	–	–	–	–	–	–	–	NA
Potassium 2-ketogluconate	–	–	–	–	–	–	–	–	–	–	NA
Potassium 5-ketogluconate	–	–	–	–	–	+	+	–	W	–	NA

[a] (Pankratov et al., 2007)
