# Supplementary material for: Mucilaginibacter florum sp. nov., isolated from the flower of Coreopsis grandiflora and Mucilaginibacter oryzagri sp. nov., isolated from rice paddy soil in Korea
Source: J Microbiol. 2025 Dec 31;63(12):e2509014. doi: 10.71150/jm.2509014 (PMC13577788; doi:10.71150/jm.2509014)
Supplement: Supplementary file 9 [file jm-2509014-Supplementary-Table-S5.pdf]

Table S5. Cellular fatty acid profiles of strains GGG–R5[T] and M4–18[T], and related type strains. Strains: 1, GGG–R5[T]; 2, M4–18[T]; 3, *Mucilaginibacter agri* KACC 21228[T]; 4, *Mucilaginibacter jinjuensis* KACC 16571[T]; 5, *Mucilaginibacter polytrichastri* KACC 19030[T]; 6, *Mucilaginibacter straminoryzae* KACC 24163[T]; 7, *Mucilaginibacter ginsenosidivorax* KACC 14955[T]; 8, *Mucilaginibacter pineti* KACC 23032[T]; 9, *Mucilaginibacter flavus* KACC 18090[T]; 10, *Mucilaginibacter dorajii* KACC 14556[T]; 11, *Mucilaginibacter paludis* TPT56[T]. Values more than 10% of the total fatty acids were bolded. –, not detected or < 1.0% of the total fatty acids.

**Fatty acids**	**1**	**2**	**3**	**4**	**5**	**6**	**7**	**8**	**9**	**10**	**11*a***
iso–$C_{15:0}$	**28.88**	**24.65**	**22.27**	**23.94**	**35.49**	**25.57**	**19.08**	**17.25**	**16.83**	**19.90**	**15.7**
anteiso–$C_{15:0}$	3.19	1.4	2.31	–	–	1.30	–	–	–	0.93	21.1
iso–$C_{15:0}$ 3OH	1.75	2.84	2.12	2.11	2.93	4.10	3.06	2.25	3	3.36	1.8
$C_{15:0}$ 2OH	–	–	1.01	–	–	1.96	–	–	–	0.43	tr
$C_{16:1}$ *ω*5*c*	2.47	6.97	3.11	2.98	2.45	2.43	4.92	6.95	1.73	2.73	5.8
$C_{16:0}$	2.72	8.16	10.02	6.45	1.09	3.79	11.93	7.15	12.65	15.10	3.1
$C_{16:0}$ 3OH	–	2.42	–	–	–	0.15	3.1	2.9	4.98	4.46	tr
$C_{17:1}$ *ω*8*c*	–	–	1.13	–	–	4.39	–	–	–	0.32	tr
iso–$C_{17:0}$ 3OH	9.3	7.47	6.32	10.99	9.63	6.94	9.43	9.42	7.34	7.50	8.9
Summed Feature 3	**32.54**	**37.76**	**36.93**	**38.88**	**34**	**29.85**	**42**	**47.08**	**46.51**	**39.10**	**24.7**
Summed Feature 9	8.25	3.19	4.83	5.87	7.71	8.83	1.81	2.99	1.98	1.19	NA

Summed feature is fatty acids that cannot be resolved reliably from another fatty acid using the chromatographic conditions chosen. The MIDI system groups these fatty acids together as one feature with a single percentage of the total. Summed feature 3 comprises $C_{16:1}$ω7c and/or $C_{16:1}$ω6c; summed feature 9 comprises of iso-$C_{17:1}$ω9c and/or 10-methyl $C_{16:0}$. tr - traces, NA - not available [a] (Pankratov et al., 2007)
