# Supplementary material for: Mucilaginibacter florum sp. nov., isolated from the flower of Coreopsis grandiflora and Mucilaginibacter oryzagri sp. nov., isolated from rice paddy soil in Korea
Source: J Microbiol. 2025 Dec 31;63(12):e2509014. doi: 10.71150/jm.2509014 (PMC13577788; doi:10.71150/jm.2509014)
Supplement: Supplementary file 10 [file jm-2509014-Supplementary-Table-S6.pdf]

Table S6. Comparison of genome characteristics of strains GGG–R5^T and M4–18^T along with their nearest phylogenetic neighbors NCBI–Genome database

Characteristics	GGG–R5^T	M4–18^T	*M. agri* R11^T	*M. jinjuensis* KACC 16571^T	*M. polytrichastri* DSM 26907^T	*M. ginsenosidivorax* KHI28^T	*M. pineti* 47C3B^T	*M. flavus* CECT 7857^T	*M. straminoryzae* RS28^T	*M. dorajii* JCM 16601^T
GenBank assembly	GCA_028736115	GCA_028736215	GCA_009928685	GCA_028596025	GCA_900116355	GCA_007971525	GCA_900101875	GCA_030409315	GCA_022953055	GCA_039536705
Assembly level	Complete	Complete	Contig	Complete	Scaffold	Complete	Contig	Contig	Scaffold	Contig
Genome size (bp)	4,809,148	7,541,737	5,598,559	6,157,393	5,813,139	7,811,413	7,200,555	6,948,697	4,759,958	7,164,473
Completeness (%)	99.07	98.74	98.11	98.26	98.55	98.45	99.03	97.58	99.32	98.74
Contigs	1	1	45	1	43	1	52	97	27	55
Contig N_{50}	4.8 Mb	7.5 Mb	791 kb	6.2 Mb	267.2 kb	7.8 Mb	365.1 kb	159.8 kb	443.8 kb	303.8 kb
Genes	4,228	6,269	4,925	5,340	5,112	6,454	6,252	5,921	4,117	5,843
Protein–coding genes	4,158	6,195	4,855	5,262	5,031	6,370	6,169	5,840	4,057	5,768
DNA G+C content (%)	41.7	43.12	42.7	42.14	42	43.07	42.91	42.41	44.5	42.5
No. of proteins	4,254	6,346	4,817	5,262	5,086	6,249	6,284	5,813	4,055	5,768
