# Supplementary material for: Mucilaginibacter florum sp. nov., isolated from the flower of Coreopsis grandiflora and Mucilaginibacter oryzagri sp. nov., isolated from rice paddy soil in Korea
Source: J Microbiol. 2025 Dec 31;63(12):e2509014. doi: 10.71150/jm.2509014 (PMC13577788; doi:10.71150/jm.2509014)
Supplement: Supplementary file 11 [file jm-2509014-Supplementary-Table-S7.pdf]

**Table S7.** Comparison of annotation statistics when using different tools to analyze genomes of strains GGG–R5$^T$ and M4–18$^T$.

| | *Mucilaginibacter* sp. GGG–R5$^T$ | | | *Mucilaginibacter* sp. M4–18$^T$ | | |
|---|---|---|---|---|---|---|
| | Prokka | RAST | NCBI Prokaryotic Genome Annotation Pipeline (PGAP) | Prokka | RAST | NCBI Prokaryotic Genome Annotation Pipeline (PGAP) |
| Predicted CDS | 4247 | 4353 | 4158 | 6341 | 6823 | 6195 |
| Predicted RNAs | 57 | 56 | 56 | 64 | 61 | 61 |
