# Supplementary material for: Mucilaginibacter florum sp. nov., isolated from the flower of Coreopsis grandiflora and Mucilaginibacter oryzagri sp. nov., isolated from rice paddy soil in Korea
Source: J Microbiol. 2025 Dec 31;63(12):e2509014. doi: 10.71150/jm.2509014 (PMC13577788; doi:10.71150/jm.2509014)
Supplement: Supplementary file 12 [file jm-2509014-Supplementary-Table-S8.pdf]

Table S8. Functional annotation of the genome of strains GGG–R5^T and M4–18^T using RAST

***Mucilaginibacter* sp. GGG–R5^T**	**hits**	***Mucilaginibacter* sp. M4–18^T**	**hits**
Amino Acids and Derivatives	181	Carbohydrates	206
Protein Metabolism	178	Amino Acids and Derivatives	202
Carbohydrates	156	Protein Metabolism	160
Cofactors, vitamins, prosthetic groups & pigments	125	Cofactors, vitamins, prosthetic groups & pigments	149
Respiration	95	Respiration	105
DNA Metabolism	65	DNA Metabolism	84
Nucleosides and Nucleotides	64	Nucleosides and Nucleotides	62
Virulence, disease & defense	47	Membrane Transport	55
Membrane Transport	45	Fatty Acids, lipids & isoprenoids	53
RNA Metabolism	43	RNA Metabolism	49
Cell Wall and Capsule	30	Virulence, disease & defense	42
Fatty Acids, lipids & isoprenoids	29	Regulation and Cell signaling	33
Phosphorus Metabolism	28	Cell Wall and Capsule	30
Stress Response	22	Stress Response	28
Regulation and Cell signaling	19	Phosphorus Metabolism	21
Metabolism of Aromatic compounds	13	Metabolism of Aromatic Compounds	13
Potassium metabolism	10	Sulfur Metabolism	13
Sulfur Metabolism	10	Potassium metabolism	12
Iron acquisition and metabolism	9	Nitrogen Metabolism	8
Secondary Metabolism	8	Miscellaneous	6
Miscellaneous	7	Secondary Metabolism	5
Nitrogen Metabolism	7	Phages, prophage, transposons & plasmids	3
Cell Division and Cell Cycle	4	Cell Division and Cell Cycle	3
Dormancy and Sporulation	1	Iron acquisition and metabolism	3
Phages, prophage, transposons & plasmids	1	Dormancy and Sporulation	1
Photosynthesis	0	Photosynthesis	0
Motility and Chemotaxis	0	Motility and Chemotaxis	0
