# Supplementary material for: Mucilaginibacter florum sp. nov., isolated from the flower of Coreopsis grandiflora and Mucilaginibacter oryzagri sp. nov., isolated from rice paddy soil in Korea
Source: J Microbiol. 2025 Dec 31;63(12):e2509014. doi: 10.71150/jm.2509014 (PMC13577788; doi:10.71150/jm.2509014)
Supplement: Supplementary file 13 [file jm-2509014-Supplementary-Table-S9.pdf]

Table S9. Functional annotation of the genome of strains GGG–R5^T and M4–18^T using RAST and eggNOG mapper

**Databases searched**	***Mucilaginibacter* sp. GGG–R5^T**	***Mucilaginibacter* sp. M4–18^T**
Total genes predicted	4,228	6,269
Protein coding genes predicted (NCBI–PGAP)	4,158	6,195
eggNOG mapper hits	3,623	5,230
GO hits	153	169
EC hits	919	1,144
PFAM hits	3,258	4,669
KEGG pathway hits	972	1,186
CAZy hits	77	113
